# Equine uveitis: Outcome and adverse effects after one or two intravitreal low‐dose gentamicin injections

**DOI:** 10.1111/evj.14056

**Published:** 2024-02-08

**Authors:** S. Morén, M. Kallberg, L. Strom

**Affiliations:** ^1^ Equine Clinic, University Animal Hospital Swedish University of Agricultural Sciences Uppsala Sweden

**Keywords:** equine ophthalmology, equine recurrent uveitis, gentamicin, horse, intravitreal injection

## Abstract

**Background:**

Uveitis is common in horses, potentially turning chronic (persistent or recurrent) resulting in impaired vision or blindness. All mainstay therapeutics aims at controlling inflammation, but long‐term or lifelong treatment is often needed with possibly severe side effects. Therefore, intravitreal injections with low‐dose gentamicin (IVGI) have been used in attempt to give a long‐lasting result with potentially less side effects.

**Objectives:**

To retrospectively assess outcome and long‐term complications following one or two low‐dose IVGI in Swedish horses with chronic uveitis.

**Study design:**

Retrospective case series.

**Methods:**

Medical records of horses diagnosed with uveitis examined at the Equine Clinic of the University Animal Hospital of Sweden between 2016 and 2021 were reviewed. Inclusion criteria were horses with a diagnosis of chronic uveitis that were treated with 4 mg IVGI. After injection, tapering doses of anti‐inflammatory medications were administered. Due to persistence or recurrence of uveitis despite IVGI, some horses received a second injection. A positive outcome was defined as controlled uveitis, despite no or minimal anti‐inflammatory medication.

**Results:**

32 eyes (29 horses) were included. Based on clinical evaluation, uveitis was classified as anterior (91%) or panuveitis (9%). 10 eyes were treated with IVGI twice. A positive outcome was observed in 13/32 eyes (41%). Inflammation in 9/32 eyes was controlled after the first, and in 4/10 eyes after the second IVGI treatment. Long‐term complications included retinal degeneration in seven eyes, and mature cataracts in five eyes. Enucleations were performed in 14/32 eyes, due to lack of favourable response of IVGI, or due to complications, that is, glaucoma, corneal ulceration, and/or corneal mineralisation. One horse was euthanised due to painful bouts of inflammation in both eyes despite treatment.

**Main limitations:**

Small sample size, retrospective design with no control group, no histopathology performed, infrequent sampling for *Leptospira* and no standardised treatment protocol after the IVGI.

**Conclusions:**

In this group of Swedish horses, predominantly diagnosed clinically with anterior uveitis, a positive outcome was observed in 41% of eyes following one or two low‐dose IVGI. Retinal degeneration in the visual streak was observed in 22% of eyes, which is a higher proportion of this complication than previously described.

## 1 BACKGROUND

Uveitis is inflammation of the uveal tract that can involve the iris and ciliary body (anterior uveitis), the choroid and retina (posterior uveitis) or involve all uveal structures (panuveitis). The disease has been described as one of the most important causes to blindness in horses, due to tendency to develop into chronic uveitis (persistent or recurrent) with permanent damage to ocular structures.^1^, ^2^, ^3^, ^4^, ^5^, ^6^ The pathophysiology of equine uveitis is complicated and has not been fully unravelled. Equine recurrent uveitis (ERU) is commonly accepted to be due to a T‐lymphocyte immune‐mediated response.^7^, ^8^, ^9^ Heterochromic iridocyclitis with secondary keratitis (HIK) is another type of chronic, persistent uveitis, which has recently been described. As in ERU, an immune‐mediated aetiology is likely, with melanosome‐related self‐antigens as the main target for the immune‐response.^10^

The aim of uveitis treatment is to control inflammation; thus, minimising pain and the detrimental effects of ongoing inflammation. However, mainstay therapeutics all have disadvantages in regards of lack of efficiency, potential severe side effects and poor compliance. This has prompted the development of surgical interventions such as pars plana vitrectomy (PPV) and Cyclosporine A suprachoroidal implants (CSI), as well as intravitreal injections.^5^, ^9^, ^11^, ^12^, ^13^, ^14^, ^15^, ^16^

In 2005, Pinard et al. proposed that *Leptospira*‐associated uveitis may respond to a low dose (4 mg) intravitreal injection of gentamicin (IVGI) and a positive outcome was seen in 17/18 eyes.^17^ Since then, more studies have been conducted, using either 4 mg or 6 mg of gentamicin intravitreally and encouraging results have been reported. Reported complications in these clinical studies were infrequent, and either no, or reduced signs of uveitis has been seen in 88%–94% of eyes.^18^, ^19^, ^20^ The mode of action of IVGI has not yet been fully elucidated. It has been proposed that gentamicin may be able to alter or suppress the action of certain T‐cell lines.^18^ However, this was not confirmed in a recent publication and further studies on the subject are warranted.^21^

In this retrospective study, the aim was to assess short‐ and long‐term results and to describe complications following one or two low‐dose (4 mg) IVGI in a case series of horses in Sweden with chronic uveitis.

## 2 MATERIALS AND METHODS

### 2.1 Cases and medical records

Medical records of horses diagnosed with chronic uveitis examined between 1 January 2016 and 30 May 2021 were reviewed. Horses fulfilling the following inclusion criteria were identified: given one or two low‐dose IVGI with a history and clinical signs consistent with chronic uveitis such as blepharospasm, epiphora, corneal fibrosis, corneal mineralisation, aqueous flare, hyalitis, miosis, iridial changes (atrophy of corpora nigra, synechia, hyper‐/hypopigmentation, fibrosis), cataractous changes, signs of retinal degeneration (presence of oedema, changes in reflectivity and/or of areas of depigmentation in the fundus), and high or low intraocular pressure (IOP). Aqueous flare was graded as 0 (none), 1 (trace), 2 (mild), 3 (moderate) or 4 (severe). Horses were excluded from the study if they had received IVGI but were lost to follow‐up. Any adverse reactions, either transient occurring within the first hours or days post‐injection (short‐term complications), or reactions occurring thereafter (long‐term complications), were noted. If enucleation was performed during the study period, time between the first IVGI until enucleation was noted.

### 2.2 Examinations and classification of uveitis

The ophthalmologic examinations were performed by a veterinary ophthalmology resident and/or a Diplomate ACVO/ECVO. Examinations included neuro‐ophthalmic evaluation, slit‐lamp biomicroscopy, tonometry and indirect ophthalmoscopy. Some horses were tested serologically for *Leptospiral* antibodies at the discretion of the attending ophthalmologist. Based on ophthalmologic examination, a diagnosis of anterior, posterior or panuveitis was made. Chronic, recurrent uveitis was diagnosed when at least two episodes of inflammation occurred after a period of quiescence following cessation of appropriate anti‐inflammatory treatment. Chronic, persistent uveitis was diagnosed when inflammation persisted for a minimum of 4 weeks despite appropriate treatment. HIK was diagnosed based on characteristic clinical signs of uveitis and corneal endothelial inflammation associated with iris pigment dispersion and retro‐corneal fibrous membrane formation.^10^

### 2.3 Intravitreal injections

All IVGI treatments were performed on standing sedated horses. Sedation was achieved using a combination of detomidine hydrochloride (0.005–0.01 mg/kg i.v.) (Domosedan vet. 10 mg/mL, Orion Pharma Animal Health) and butorphanol (0.005–0.01 mg/kg i.v.) (Butomidor vet. 10 mg/mL, Salfarm Scandinavia). Local akinesia and analgesia were achieved through blocks to the auriculopalpebral and supraorbital nerves with mepivacaine (1–1.5 mL/injection site s.c., Mepidor Vet. 20 mg/mL, Salfarm Scandinavia), and by topical application of oxibuprocaine at the site of injection (Oxibuprokain Bausch & Lomb 0.4%, Bausch & Lomb). The conjunctival fornices were rinsed thoroughly with 20 mL 0.05% chlorhexidine solution and 20 mL of balanced saline solution prior to injection. Topical phenylephrine (Phenylephrin hydrochloride 10%, Bausch & Lomb) was applied at the site of injection to minimise bleeding. A nose twitch was applied if deemed necessary to facilitate the injection being performed safely. The injection was performed at the 12 o'clock position on the globe, 10 mm posterior to the limbus as previously described by Fischer et al.^18^ For easier access to the dorsal sclera, the horse's head was rotated to produce a ventral globe rotation. Using a 1 mL insulin syringe (29‐gauge, 12.7 mm long, BD Micro‐Fine), sterile intravitreal injections were performed with 4 mg of preservative‐free gentamicin in 30 eyes (0.1 mL Gensumycin 40 mg/mL, Sanofi AB), and with 4 mg of gentamicin containing preservatives (0.04 mL Gentaject® vet. 100 mg/mL, Ceva Animal Health) in 2 eyes. Indirect ophthalmoscopy was performed immediately after the injection to identify any intracameral and intravitreal haemorrhage.

### 2.4 Post‐injection therapy

Post‐injection therapy consisted of topical corticosteroids (dexamethasone, Isopto‐Maxidex 0.1%, q8–12h, Novartis, or prednisolone pivalate, Ultracortenol 0.5%, q12h, Agepha Pharma s.r.o.), and systemic treatment per orally with either NSAIDs (flunixin meglumine, Flunipaste, 0.5–1.1 mg/kg q12–24h, Bio Vet, or meloxicam, Metacam, 0.6 mg/kg q24h, Boehringer Ingelheim Animal Health) or corticosteroids (prednisolone, Equisolon, 0.25–1 mg/kg q12–24h, Dechra Veterinary Products). Most horses also received topical mydriatics (atropine, Isopto®‐Atropine 1%, q12–48h, Alcon Nordic). At follow up examinations, the anti‐inflammatory medications were adjusted and tapered based on prevailing clinical signs and response to treatment (Table 1).

**TABLE 1:** overview of all patients treated with low‐dose IVGI, post‐injection therapy, complications and outcome.

Case	Breed, gender, age	Eye	Diagnosis	Leptospiral testing	Topical treatment (months or C = continous)	Systemic treatment (months or continous)	Numbers of IVGI	Follow up time (days)	Glaucoma	Complications	Enucleation/euthanazia	Outcome first injection	Outcome second injection	
1	Friesian horse, G, 11	OD	AU, ERU	No	Pred, 5	Flun, 2	1	485	No		‐	P	‐	
2	Crossbreed, M, 21	OD	AU, ERU	Negative	Dex, 2	Mel, 1	2	335	No	U (d335), M	Enucl	P	N	P until ulceration d335, no response to IVGI 2
3	STB, G, 20	OS	AU, ERU	No	Dex, 2	Flun, C	1	42	Yes (pre‐injection)		Enucl	N	‐	
4	BWP, M, 8	OS	AU, HIK	No	Dex, C	Mel, 1	1	90	No	M, RD	‐	N	‐	
5	Hanoveranian, G, 14	OS	AU, ERU	Positive	Dex, C	Mel, 2	1	123	No	M, RD	Enucl	N		
6	SWB, G, 10	OD	AU, HIK	No	Dex, C	Flun, C	2	76	No		Enucl	N	N	
7	North Swedish Horse, G, 22	OD	AU, HIK	No	Dex, C	Mel, C	1	56	No		Enucl	N	‐	
8	Crossbreed, G, 13	OD	AU, ERU	Negative	Dex, 1	Flun, 1	1	531	No		‐	P	‐	
9	KWPN, M, 13	OS	AU, ERU	Negative	Dex, 6	Flun, 5	2	210	No	RD	‐	N	P	
10	Welsh cob, M, 9	OS	AU, HIK	Negative	Pred, C	Flun, 4	2	378	No	U	‐	N	N	
11	SWB, M, 8	OS	AU, ERU	Negative	Dex, C	Flun, 1	1	371	No	U, M	‐	N	‐	
12	KWPN, G, 8	OS	AU, HIK	No	Dex, 2	Mel, 3	1	161	No	U	‐	P	‐	
13	Crossbreed, M, 12	OD	AU, HIK	No	Dex, 7	Mel, C	2	197	No		Enucl	N	N	
14	KWPN, M, 7	OS	AU, ERU	No	Dex, C	Flun, 4	1	155	No		Enucl	N	‐	
15	Appaloosa, G, 9	OD	AU, ERU	No	Pred, C	Pred, 2	1	238	No	RD, U, M, C	Euth	N	‐	
16	Appaloosa, G, 9	OS	AU, ERU	No	Pred, C	Pred, 2	1	238	No	RD, U, M, C	Euth	N	‐	
17	SWB, M, 16	OD	AU, ERU	No	Dex, 2	Flun, 2	1	462	No		‐	P	‐	
18	STB, G, 4	OD	AU, HIK	No	Dex, 2	Flun, 1	1	35	No		‐	P	‐	
19	SWB, M, 13	OD	AU, HIK	No	Dex, C	Flun, C	2	155	No		Enucl	N	N	
20	SWB, G, 10	OD	AU, ERU	No	Dex, 2	Flun, 2	2	415	No	U, M	‐	N	P	
21	AQH, G, 10	OD	AU, HIK	No	None, 0	Pred, 2	1	84	No		‐	P	‐	
22	Crossbreed, M, 12	OD	AU, ERU	No	Dex, C	None, 0	2	184	Yes (pre‐injection)	U, M	Enucl	N	N	
23	SWB, G, 13	OD	AU, HIK	No	Pred, 3	Mel, 2	1	91	No	U	Enucl	P	‐	
24	SWB, G, 19	OD	PU, ERU	No	Dex, C	Flun, 2	1	567	Yes (post‐injection)	U	‐	N	‐	
25	SWB, G, 19	OS	PU, ERU	No	Dex, C	Flun, 2	1	196	Yes (pre‐injection)		Enucl	N	‐	
26	SWB, G, 21	OD	AU, ERU	No	Dex, C	None, 0	1	229	Yes (post‐injection)	U, M	Enucl	N	‐	
27	Polo pony, M, 13	OD	AU, ERU	No	Pred, 2	Mel, 4	2	440	No	C	‐	N	P	
28	Polo pony, M, 13	OS	AU, ERU	No	Pred, 2	Mel, 4	2	440	No	C	‐	N	P	
29	SWB, M, 10	OD	AU, ERU	No	Dex, 1	Flun, 1	1	728	No	C	‐	P	‐	
30	KWPN, G, 10	OD	AU, ERU	Negative	Dex, C	Flun, 2	1	278	No	RD, U, M	Enucl	N	‐	
31	Knabstrup, M, 23	OD	PU, ERU	No	Dex, C	Mel, C	1	45	No		Enucl	N	‐	
32	SWB, M, 14	OD	AU, HIK	No	Dex, C	Mel, 2	1	286	No	U, M, RD	‐	N	‐	

*Note*: Breed: AQH, American Quarter Horse; BWP, Belgian Warmblood; KWPN, Dutch Warmblood; STB, Standardbred Trotter; SWB, Swedish Warmblood. Gender: G, Gelding; M, Mare. Diagnosis: AU, Anterior Uveitis; PU, Panuveitis. Treatments: Dex = Dexamethasone; Flu = Flunixine; Mel = Meloxicam; Pred = Prednisolone. Complications: C, Cataract; G, Glaucoma; M, Mineralisation; RD, Retinal degeneration; U, Ulceration. Outcome: N, negative; P, positive.

### 2.5 Follow‐up examinations

Follow‐up examinations were performed within 1–2 weeks and at 1‐month post‐injection. Thereafter, further examinations were scheduled individually for each patient, based on the progression and development of remaining clinical signs.

### 2.6 Positive outcome

A positive outcome was defined as no, or markedly reduced, signs of uveitis (comfortable eye, no or trace of aqueous flare, normalised or close to normalised IOP) despite no or minimal anti‐inflammatory treatment (i.e., flunixin meglumine p.o. 0.25–0.5 mg/kg once daily or topical dexamethasone/prednisolone once daily‐every other day). To be classified as a positive outcome despite intermittent or continuous low‐grade anti‐inflammatory medication, the dosage of medication also had to be lower than before IVGI.

## 3 RESULTS

**FIGURE 1:**
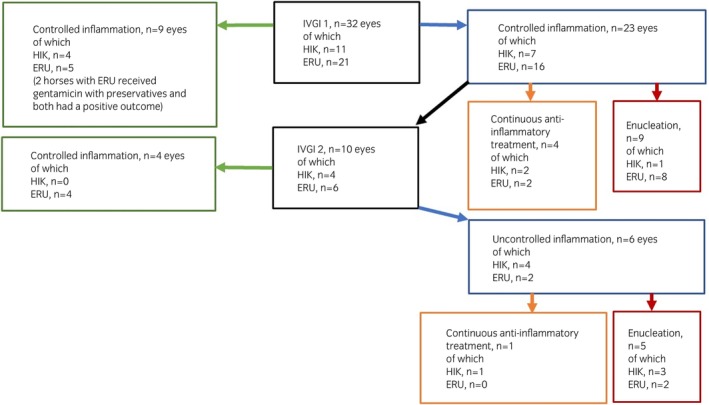
Flow chart describing clinical outcome and further treatment of 32 eyes (29 horses) with uveitis. ERU, equine recurrent uveitis; HIK, heterochromic iridocyclitis with secondary keratitis; IVGI 1, one intravitreal injection of gentamicin (4 mg); IVGI 2, second intravitreal injection of gentamicin (4 mg).

The inclusion criteria were met in a total of 32 eyes (29 horses). Uveitis was classified as anterior (91%) or panuveitis (9%). None of the horses were diagnosed with posterior uveitis only. In total, a positive outcome was observed in 13/32 eyes (41%) after one or two IVGI (Figure 1). Median follow‐up time for all eyes was 220 days (range 35–728). Median follow‐up time was 139 days (range 35–728) after the first injection, and 137 days (range 33–350) after the second injection. Mean age was similar when groups with positive and negative outcomes were compared (12 vs. 13 years). 6/13 eyes with positive outcome were controlled without ongoing medication, 2/13 eyes received minimal treatment intermittently (i.e., dexamethasone topically once daily or every other day during winter, and no medication during summer), and 5/13 eyes in the positive outcome group received continuous minimal anti‐inflammatory medication (i.e., flunixin meglumine p.o. 0.25–0.5 mg/kg once daily or topical dexamethasone/prednisolone once daily‐every other day).

Chronic recurrent or persistent uveitis (ERU) was diagnosed in 21/32 eyes (66%), and a positive outcome was observed in 5/21 (24%) of these eyes after one IVGI. HIK was diagnosed in 11 of 32 eyes (34%), and a positive outcome was observed in 4/11 (36%) of these eyes after one IVGI. 10 eyes (6 ERU, 4 HIK) received a second IVGI (median time post first injection: 80 days, range 43–378) due to either persistence of inflammation despite a first IVGI and concurrent anti‐inflammatory medication, or recurrence of inflammation when the anti‐inflammatory medication was progressively reduced. After the second IVGI, a positive outcome was observed in 4/6 eyes (67%) with ERU, whereas none of the 4 eyes with HIK responded to the second treatment.

### 3.1 Leptospira

In total 7 of 29 horses were tested serologically for leptospirosis: 1/7 was positive for *Leptospira (L.)*, *L*. Grippotyphosa. This horse received one IVGI and did not respond to treatment.

### 3.2 Complications

#### 3.2.1 Peri‐injection complications

Peri‐injection complications occurred in 5/32 eyes (16%). Adverse reactions included mild ocular discomfort (3/5), mild periocular swelling and mild to severe serous epiphora (4/5), full body urticaria (1/5), and ventromedial strabismus (1/5). The ventromedial strabismus resolved in <2 months, all other complications resolved within 1 week.

#### 3.2.2 Long‐term complications

Long‐term complications included retinal degeneration, mature cataract, corneal mineralisation, and corneal ulcers. Adequate evaluation of the ocular fundus was possible in all eyes immediately before and after the IVGI, but not in all eyes at all follow‐up examinations due to progressive cataract formation.

**FIGURE 2:**
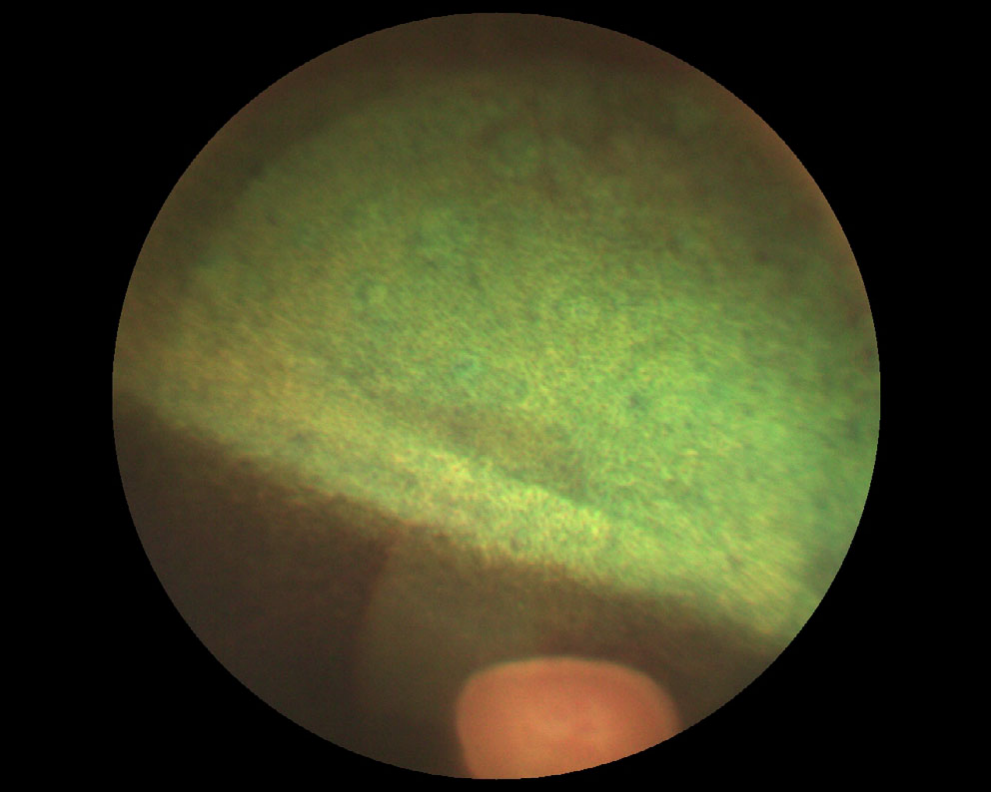
10 year old Dutch Warmblood gelding with equine recurrent uveitis in the right eye: 14 days following intravitreal injection of 4 mg gentamicin, a horizontal area of diffuse tapetal hyperreflectivity in the area of the visual streak with most distinct abnormalities in the area centralis was detected during indirect ophthalmoscopy.

1. Retinal degeneration was diagnosed post‐injection in 7/32 eyes (22%, 5 eyes with ERU and 2 eyes with HIK, all injected with preservative‐free gentamicin) and was observed 14 days (3/7 eyes), 42–44 days (3/7 eyes), or 161 days (1/7 eyes) after the IVGI. None of the eyes had been diagnosed with fundus abnormalities pre‐IVGI. A horizontal area of diffuse tapetal hyperreflectivity in the area of the visual streak with most distinct abnormalities in the area centralis developed in all eyes diagnosed with retinal degeneration (Figure 2). All but 1 eye received one IVGI, and 6/7 eyes had a negative outcome. The 1 eye that received two injections had a positive outcome in regards to control of uveitis, but developed retinal changes (retinal oedema) within a week after the second IVGI and retinal degeneration was confirmed after 44 days.
2. Mature cataracts developed in 5/32 eyes (16%, 3 horses, all eyes with ERU, all injected with preservative‐free gentamicin). 2 of these horses (3 eyes) had slight cataractous changes (focal, incipient cataracts) before injection and developed mature cataracts 420 (1 eye) and 350 days (2 eyes) after the first and second injection respectively. These 3 eyes had a positive outcome in regards of control of uveitis: 1 after a single IVGI and 2 after receiving a second IVGI. The third horse (2 eyes) had no lens abnormalities, except for slight pigment dispersion on the lens capsule before IVGI, but developed total cataracts in both eyes 210 and 238 days after one IVGI, and ulceration and mineralisation of the cornea occurred concurrently. Both of this horse's eyes were considered to have a negative outcome.
3. Corneal mineralisation occurred in 12/32 eyes (38%), at a median time of 160 days (range 14–370 days) post first injection (10 eyes) and in 44 and 114 days post second injection (2 eyes). 1 of these eyes had slight mineralisation pre‐injection that became more marked after IVGI. 7/12 eyes were treated with topical dexamethasone and 1/12 eyes with topical prednisolone at the time of mineralisation. Of the eyes developing corneal mineralisation, 3/12 were classified as having a positive outcome in regards of control of uveitis after IVGI, and were not receiving continuous topical treatment, but corneal mineralisation developed 14, 44 and 370 days after the IVGI respectively.
4. Corneal ulceration occurred in 13/32 eyes (41%) at a median time of 210 days (range 14–370 days) post first injection (10 eyes), and at a median time of 106 days (range 44–267 days) post second injection (3 eyes). 12/13 eyes were treated with topical corticosteroids at the time of ulceration. 3/13 eyes were classified as having a positive outcome in regards of control of uveitis (2 after one IVGI and 1 after a second IVGI), but developed corneal ulcers 14, 44 and 370 days after the last IVGI respectively.

### 3.3 Glaucoma

Glaucoma was diagnosed in 3/32 eyes (9%, all ERU eyes) before IVGI. None of these eyes responded to treatment and all were enucleated. 2 of the eyes (both ERU eyes) developed glaucoma after the IVGI; 1 eye responded to increased doses of anti‐inflammatory and anti‐glaucoma medication (i.e., dorzolamide and timolol in a combination preparation, topically q8h) (Cosopt 20 + 5 mg/mL, Santen Oy), the other eye did not respond to treatment and was enucleated.

### 3.4 Enucleations

Enucleations were performed in 14/32 eyes (44% of eyes; 9/21; 43% of eyes with ERU and 5/11; 45% of eyes with HIK) at a median time of 109 days after the last IVGI (range 34–547 days). Enucleation was performed due to increased inflammation in spite of anti‐inflammatory medications and/or due to complications such as glaucoma, corneal ulceration and/or corneal mineralisation.

## 4 DISCUSSION

A positive outcome was observed in 28% (9/32, five ERU and four HIK eyes) of the eyes after one intravitreal injection with a low dose of gentamicin in this group of horses predominately diagnosed with anterior uveitis. A second IVGI in 10 eyes, resulted in control of inflammation in an additional 4 eyes (only ERU eyes). However, only 13/32 of the eyes (41%) responded to one or two IVGI in regards of control of uveitis. In addition, in 13 of the eyes classified as having a positive outcome, only 6/13 could stop concurrent medication completely. Of the positive outcomes, 2/13 were treated intermittently and 5/13 were treated continuously with anti‐inflammatory medication, although at a reduced frequency than before the IVGI. Nearly half of the eyes (14/32, 44%) were enucleated due to lack of response to treatment and/or due to complications to concurrent medical treatment. 1 horse was euthanised after developing retinal changes and thereafter mature cataracts in both eyes post IVGI.

The lower success rate in the current study compared with other reports, where a positive outcome has been described in 88%–94% of treated eyes,^17^, ^18^, ^19^, ^20^ may be due to several factors. A different case‐load with horses mainly suffering from anterior uveitis (91%) was included in this study, and there may be a lower prevalence of leptospirosis as an inciting factor for ERU in Swedish horses. Also, in some cases the IVGI in the current study might have been performed too late in the course of disease and it is possible that if advanced concurrent disease of the eye, such as glaucoma, has already developed, the eye may not respond well to low‐dose IVGI. Five eyes were injected as a last attempt to avoid enucleation and IVGI was unsuccessful in all these eyes. Additionally, the 3 eyes with pre‐existing glaucoma were also enucleated. None of pre‐injection glaucoma eyes were rescued by this treatment in the present study.

In earlier equine uveitis studies, the prevalence of anterior uveitis has been reported to be between 10% and 20% whereas it was 91% in this study.^18^, ^19^ The injections were performed in the vitreous and it is known that barriers in the eye prevent diffusion throughout the eye.^22^, ^23^ This may explain why many of the horses in the present study responded poorly to the IVGI, compared with horses in other studies mainly describing cases of pan‐ or posterior uveitis.^18^, ^19^ In humans, there are more than 30 different classifications of uveitis depending on where inflammation is most severe, and treatment may differ between different forms of uveitis.^24^ Intravitreal injections are most often used as a treatment for pan‐ or posterior uveitis.^25^, ^26^, ^27^, ^28^ In this study, there were only a few cases that showed signs of inflammation in the posterior segment, that is, the site where the injections were administered. Injection of gentamicin into the anterior segment has not yet been studied in horses, and we do not know if this route of administration would lead to a higher rate of success with less adverse effects in anterior uveitis, but potential unwanted side effects must also be considered. Peyman et al. (1974) showed that no residual damage to ocular structures was seen when injecting up to 8000 μg of gentamicin in the anterior chamber in rabbits, unlike when the same dose was administered as an intravitreal injection. The authors also concluded that gentamicin has a faster clearance from the anterior chamber than from the vitreous.^22^ On the other hand, in another study also performed on rabbit eyes, injections of 1000 μg of gentamicin in the anterior chamber caused endothelial damage.^29^ In future studies it may be interesting to study injections of gentamicin into the anterior chamber in horses with chronic, anterior uveitis to evaluate the outcome in regards of effect compared with IVGI. However, toxicity to the endothelium when performing intracameral injections of gentamicin in the horse must also be considered and evaluated.

Leptospirosis is a well‐known inciting factor for chronic, persistent or recurrent uveitis.^12^, ^30^, ^31^, ^32^ The disease is endemic in many parts of the world, particularly in moist tropical and subtropical areas.^30^, ^33^ Baverud et al. (2009) reported a seroprevalence of 25% in Swedish horses, but almost never related to clinical disease.^34^ *L*. Pomona and *L*. Grippotyphosa are described as a common causative agent to ERU,^31^, ^32^ but these serovars were only prevalent in 0.4% and 0.5% respectively of the horses in the study by Baverud et al.^34^ From 2001 to 2021, 0–3 reported cases of seropositive horses were reported to the Swedish Board of Agriculture each year, and the prevalence of leptospirosis in Sweden is probably low.^35^ Therefore, leptospiral testing has not previously been included in the standard protocol in the evaluation of Swedish horses with uveitis. In the current retrospective study, serological sampling was only performed in 7/29 horses with only 1 imported horse being positive for antibodies (*L*. Grippotyphosa), and no eyes were subjected to aqueous or vitreous testing. Thus we cannot draw any conclusions on whether the leptospira status could have been an important factor to influence the results. Further studies to evaluate leptospirosis as a potential initiating factor for chronic uveitis in Swedish horses should be performed.

A higher prevalence of retinal degeneration was observed in this study, compared with previously described rates: we noted retinal degeneration in 22% of treated eyes post IVGI, and both ERU and HIK eyes were affected. No signs of retinal degeneration had been seen in these eyes pre‐injection. Fischer et al. (2019) report this complication in 5.1% of injected eyes whereas Launois et al. (2019) observed an increase in the prevalence of retinal changes from 28% to 36% in eyes before and after injection respectively.^18^, ^19^ The current study is small and does not include a control group, thus it is difficult to conclude that the injection itself caused the retinal change. However, the retinal abnormalities appeared within weeks post‐IVGI (within 2 weeks in 3/7 eyes and within 8 weeks in 3/7 eyes) and therefore, toxicity from the gentamicin injection may be a reasonable cause. Antibiotics injected intraocularly are known to induce detrimental effects, and toxicity of gentamicin administered to the eye has been demonstrated in humans, rabbits, monkeys and pigs.^22^, ^23^, ^36^, ^37^ In an experimental study on rabbits, doses above 500 μg gentamicin injected intravitreally induced many toxic side effects and retinal degeneration and mature cataracts were most common. When injected with 1000 μg, 50% of rabbits developed retinal degeneration, and when injected with 2000–8000 μg, 100% developed retinal degeneration.^22^ The rabbit vitreous contains approximately 1.4 mL of fluid,^22^ whereas on average, the equine vitreous contains about 28 mL.^38^ Based on the study of Peyman et al. (1974), a non‐toxic concentration of gentamicin injected intravitreally in rabbit eyes is approximately 360 μg/mL.^22^ In comparison, a low dose (4 mg) of gentamicin injected intravitreally in an equine eye will result in a concentration of approximately 140 μg/mL. However, it is not known if horses may be more sensitive than rabbits to the toxic intravitreal effects of gentamicin.

To evaluate gentamicin toxicity after intravitreal injections in monkeys, histopathology was performed in a study by Brown et al. (1990) which concluded that the retinal changes were mainly neural in the early stages (hours) after injection, and mainly vascular in the later stages (<11 days) with thrombi‐formation in retinal vessels and subsequent vascular and retinal necrosis.^37^ Potentially, the same toxic changes could occur after IVGI in horses. It may be one explanation to why some horses in this study showed signs of impaired vision and behavioural changes after the IVGI, without obvious other causes such as cataract formation, and before abnormal findings were observed on ophthalmoscopy. In the current retrospective study, retinal changes were observed by ophthalmoscopy and histopathology was not performed on enucleated eyes. In further studies, it would be of great value to perform histopathologic studies of equine eyes after IVGI to evaluate the presence and extent of toxic effects. Also, future studies should include pre‐ and post‐recordings of electroretinograms (ERGs) to assess retinal function before and after the IVGI, with the aim to explore the potential toxic effects on retinal cells and retinal function, and thus to evaluate impact on visual performance.

In this study, 5/32, 16% of eyes developed mature cataracts, which is comparable to 8.5% and 12% in other studies on IVGI.^18^, ^19^ All cataracts progressed in about 6–12 months, or in one eye, >12 months after injection.^22^ Uveitis itself can induce cataracts,^1^, ^2^, ^3^ so although cataracts may have been induced by the injection, it is also likely that the cataracts developed due to persistent, or previous, episodes of uveitis.

Many of the horses included in this study experienced recurrence of uveitis when developing corneal mineralisation and sometimes subsequent ulceration of the cornea during the follow up period. Topical corticosteroids, and continuous intraocular inflammation, can disrupt calcium homeostasis with secondary dystrophic mineralisation of the cornea, and there are also other severe complications described due to long‐term continuous treatment with corticosteroids such as secondary ocular infection following suppression of host response.^39^, ^40^ Some of the horses were given continuous topical anti‐inflammatory treatment long after the IVGI due to signs of low‐grade ocular inflammation, based on continuous low IOP, trace aqueous flare and/or low‐grade miosis. In these horses, it is impossible to know whether the inflammation itself, the treatment, or both contributed to corneal mineralisation and subsequent ulcers. 3 eyes classified as having a positive outcome at initial re‐check, later developed corneal mineralisation and/or ulcers and thereafter recurrence of uveitis. It is a possibility that unnoticed persistent or recurrent uveitis were the cause of these complications.

Although a low number of cases with a positive outcome and a high number of long‐term complications was reported in this retrospective case series, the injection procedure was generally well tolerated by the horses. Peri‐injection complications were few (5/32 eyes, 16%) and mainly consisted of mild peri‐ocular swelling, epiphora and discomfort which all resolved within a couple of days. One horse developed generalised urticaria after the injection, which resolved completely within 1 week. This adverse advent may have been due to a reaction to the IVGI, or more likely relate to the drugs given for sedation or local anaesthetics. 1 horse developed ventromedial strabismus that resolved within 2 months. This complication was unexpected and we hypothesise some sort of impact on extraocular muscles and/or cranial nerves due to the injection itself. It is unlikely that the nerves and muscles controlling the eye position were affected by the supraorbital and palpebral nerve blocks applied before the injection, and the only other plausible explanation would be that this occurred due to a complication to the intravitreal injection itself and thus slight trauma to the globe and periocular structures.

In conclusion, in this group of Swedish horses predominantly with a clinical diagnosis of anterior uveitis, a positive outcome was observed in 41% of eyes following one or two low‐doses of IVGI. In eyes with ERU, 26% responded to the first injection, and a positive outcome was observed in 67% of eyes subjected to a second injection. In eyes with HIK, 36% responded to the first injection, and none showed a positive outcome after a second injection. Retinal degeneration in the visual streak was observed in 22% of eyes, which is a higher proportion of this complication than previously described. In future studies it would be of great value to perform histopathology on enucleated eyes, as well as ERGs before and after IVGI, to be able to assess retinal function and potential toxic effects. It would also be interesting to investigate whether gentamicin injection in the anterior chamber could improve outcome and minimise detrimental effects in cases of anterior uveitis but the potential toxicity to the corneal endothelium must also be considered.

## AUTHOR CONTRIBUTIONS

**S. Morén:** Writing – original draft; writing – review and editing. **M. Kallberg:** Supervision. **L. Strom:** Supervision; writing – review and editing.

## FUNDING INFORMATION

Not applicable.

## CONFLICT OF INTEREST STATEMENT

The authors have no competing interests to declare.

## DATA INTEGRITY STATEMENT

S. Morén had full access to all data in the study and takes responsibility for the integrity of the data.

## ETHICAL ANIMAL RESEARCH

No ethical approval needed for this retrospective study.

## INFORMED CONSENT

Not applicable.

### PEER REVIEW

The peer review history for this article is available at https://www.webofscience.com/api/gateway/wos/peer-review/10.1111/evj.14056.

## Data Availability

The data that support the findings of this study are available from the corresponding author upon reasonable request. Data sharing exemption granted by the editor for this retrospective case series.
